# Scraps

**Published:** 1888-12

**Authors:** 


### SCRAPS

PICKED UP BY THE ASSISTANT-EDITOR.

---

**Concentrated.**—A Vassar graduate, out in the country, went into the stable of a farm-house. "Dear me; how close the poor cows are crowded together!" she remarked. "Yes, mum; but we have to do it." "Why so?" "To get condensed milk, mum." The sweet girl graduate went off relieved and enlightened.

**School Anatomy.**—The following was recently turned in as a *boná fide* composition by an Indiana schoolboy: "The human body is made up of the head, the thorax and the abdomen. The head contains the brains, when there is any. The thorax contains the heart, lungs, and diafram. The abdomen contains the bowels of which there are five, A, E, I, O, U, and sometimes W and Y."—*The Medical and Surgical Reporter*.

**Better than Nothing.**—A very handsome man, of large form, fell off the railway platform at Camp Hill, falling under the train, and had one thigh and the leg of the other below the knee completely smashed. He was brought to the General Hospital, and both limbs were at once amputated. The case gave me much anxiety; but he continued to live, the wounds healing well. I found he was engaged to be married to a very nice, respectable young woman, whom I found one evening sitting by his side. I said I thought she should think very seriously about her engagement, hinting that I thought it had better be broken off; but she most naïvely said, "Oh! sir, I am quite content to take the rest of him."—Mr. Dickinson Compton in *Guy's Hospital Reports*.

**Misconception.**—The following verses must have been written in ignorance of the limitations which medical women set themselves in the practice of their profession:

Oh, she comes in silk and satin;
She is versed in roots of Latin,
As well as every root that grows below the mother-earth;
She reads Sanskrit, she reads Coptic;
She's the apple of my optic;
Of degrees she has a lengthy list, and Girton saw her birth!

Yet it sets my blood ashiver
When she asks about my liver;
And I stutter and am speechless when my tongue she wants to see,
For I'm fearful to expose it
In *négligé*—she knows it,
When her lovely eyes with tender light are riveted on me.

With my pulses at a hundred,
'Tis not strange that she has blundered,
And doctored me for fevers when I didn't have a sign;
'Tis her presence that is heating,
And which sets my heart a-beating,
For I'm only a poor mortal man, and she is so divine!

Still it's pleasant to be ailing,
Just to have an angel sailing
Into your humble room and fill it full of life and light;
But, as for a diagnosis,
Why, what anybody knows is
Impossible at such a time to formulate aright.

**Doctors' Fees.**—In *The Medical Age* (Detroit, U.S.A.) there has been a symposium on this question. Dr. F. J. Groner says: "When the practice of medicine is the business of a man's life, it is just as legitimate as any other business. It is no more proper for a doctor to do a long-credit business, or a dead-beat business, than it is for men in any other field. The first great duty, of course, is 'to answer the cry of humanity.' The second great duty is to promptly send in your bill. . . . If you do not hear from your patron, promptly send him another reminder, or call upon him personally. By this means you will very soon find out whether he intends to pay the bill, or whether he belongs to a large class who never pay a doctor. There is too much sentimental nonsense in the idea that a physician must be an unselfish, devoted humanitarian, giving up the strong years of his life running after dead-beats. Business is business. . . . No true physician will refuse to do genuine charity work, or to extend credit to the worthy poor. Someone has divided the poor into three classes —the Lord's poor, the devil's poor, and the poor devils. The first and last are worthy objects of charity. The less you have to do with the devil's poor the better you are off. Never doctor them to have their good-will, or to have them say you are a good doctor. Sooner or later they will curse you. Give them to understand that your terms with them are cash. They will respect you, and when they get very sick will have money for you. . . . Physicians should unite and form protective associations, and refuse to extend credit to any party found on the delinquent list, until the old accounts are settled." (See *Journal*, p. 151.)

**What are we to Know ?**—Canon Isaac Taylor, in an address on "Literature and Culture," has given some advice which, with some obvious modifications, doctors and medical students would do well to lay to heart. He said: "It is impossible for any one man to know everything—*omne scibile.* Two hundred years ago it was possible; but now the domain of knowledge has been so vastly extended that it is impossible. In these days, to be distinguished a man must be a specialist—he must devote himself to some department of knowledge. It is impossible, for instance, that the same man can be at once a great historian and a great chemist. It was said of Whewell, that while science was his forte, omniscience was his foible. Because he knew so much, he thought he could know everything; and so, if you strive after universal knowledge, what you will attain will not be science, but sciolism. Strive after universal knowledge, and you will become a universal smatterer. On the other hand, the mere specialist tends to become a narrow pedant. How shall we avoid these opposite dangers—sciolism and pedantry ? I think the best rule for a young man to set before him in his studies is, to resolve to know something of everything, and everything of something. To know everything of something is to choose some one branch of science for your speciality, and to learn everything that can be learned about it: this will give you accuracy and precision. To become 'an authority,' as it is called, on some one subject, however small—on butterflies, birds' eggs, or even on postage stamps—is better than nothing. Still better is it to take some department of history, or of geology, or of philology, and master it thoroughly. But this is not enough. You will thus learn to be accurate, exhaustive; but you will be narrow. You must not only know everything of something; but, to gain breadth of mind, you should also resolve to know something of everything. Strive to take a general interest in every department of human knowledge. Strive to know enough of every science to enable you to listen intelligently to any great specialist you may chance to meet, and to read with profit any great epoch-making book that may appear. Such knowledge cannot be deep; but such an imperfect knowledge adds immensely to the intellectual pleasures within your reach, and will constantly throw light on that one branch of knowledge which you have specially made your own."—*The Church-Worker.*

